# The benefits of modified FOLFIRINOX for advanced pancreatic cancer and its induced adverse events: a systematic review and meta-analysis

**DOI:** 10.1038/s41598-018-26811-9

**Published:** 2018-06-06

**Authors:** Hongxuan Tong, Zhu Fan, Biyuan Liu, Tao Lu

**Affiliations:** School of Life Sciences, Beijing University of Chinese Medicine, Beijing, 10029 China

## Abstract

FOLFIRINOX has been one of the first-line options for advanced pancreatic cancer, even though it induces significant adverse effects. Several institutions have begun using modified FOLFIRINOX to decrease its side effects and increase its tolerability. We systematically investigated the outcome from patients who initially received modified FOLFIRINOX as a chemotherapy regimen for advanced pancreatic cancer. We used the random-model generic inverse variance method to analyse the binary data with 95% confidence intervals (CIs). Eleven studies were included in the meta-analysis with 563 total patients. The 6-month and 1-year overall survival (OS) rates of locally advanced pancreatic cancer (LAPC) were 90.9% and 76.2%. The 6-month and 1-year progression-free survival (PFS) rates of LAPC were 81.5% and 48.5%. The 6-month and 1-year OS rates of metastatic pancreatic cancer (MPC) were 79.7% and 47.6%. The 6-month and 1-year PFS rates of MPC were 56.3% and 20.6%. The following rates were also calculated: complete response rate (CR): 2.9%; partial response rate (PR): 35.9%; stable disease rate (SD): 41.2%; overall response rate (OR): 34.6%; disease control rate (DCR): 76.7%; progressive disease: 23.1%; and grade III/IV adverse events (AEs): neutropenia 23.1%, febrile neutropenia 4.8%, thrombocytopenia 4.8%, anaemia 5.7%, fatigue 11.5%, nausea 9.1%, diarrhoea 10.1%, vomiting 5.7%, neuropathy 3.8%, and increased ALT 5.7%. In conclusion, modified FOLFIRINOX could provide comparative survival benefits with fewer adverse events compared to the conventional dosage.

## Introduction

Pancreatic cancer (PC) has one of the highest cancer mortality rates in the world^1^. In 2017, the estimated number of deaths from pancreatic cancer was 43,090 in the United States; further, the 5-year relative survival rate was only 8%, and that of the distant stage was only 3%^2^. Pancreatic cancer is currently the third leading cause of cancer-related deaths in the United States^3^ and will become the second leading cause in 2030^4^. Because most cases are diagnosed at late stages as either metastatic or locally advanced^5–8^, curative surgical resection can be performed in only 15–20% of cases^9,10^.

Other than surgical resection, systemic chemotherapy is the only major treatment that can improve survival for patients with locally advanced or metastatic pancreatic cancer. Twenty years ago, gemcitabine (GEM) replaced 5-fluorouracil (5-FU) as the main chemotherapeutic drug for treating advanced pancreatic cancer because a modest survival increase (5.65 vs 4.41 months) and more clinical benefits were found in a Phase III clinical trial^11^. Since then, gemcitabine monotherapy had been the gold standard for pancreatic cancer. Later, numerous clinical trials combined gemcitabine with other anti-tumour agents to increase the anti-tumour effects, but most such studies were unable to demonstrate the superiority of or a significant improvement in OS for gemcitabine combination therapy; only gemcitabine combined with capecitabine and erlotinib have shown promise^5,6,12,13^.

Recently, in the PRODIGE 4/ACCORD 11 randomized trials, a four-drug regimen called FOLFIRINOX, consisting of folinic acid, 5-fluorouracil, irinotecan and oxaliplatin, was demonstrated to prolong overall survival compared to gemcitabine monotherapy (11.1 months vs 6.8 months). These results suggested that this combined regimen should be used in clinical practice as a first-line option for advanced pancreatic cancer patients^14^. Shortly thereafter, a regimen of gemcitabine and albumin-bound paclitaxel was shown to have statistically significant survival benefits in OS and PFS, thus providing another choice for treating advanced pancreatic cancer^15^. However, FOLFIRINOX appears to be more effective than GEM/NAB-P^16^. Although FOLFIRINOX is a first-line option for patients with metastatic pancreatic cancer, there is a controversy about whether the survival benefits of the four-drug combination regimen outweigh the associated toxicities^17^. The significant adverse effects induced by this regimen include neutropenia, thrombocytopenia, febrile, diarrhoea neutropenia, febrile neutropenia, thrombocytopenia, diarrhoea, and neuropathy, which limit its usage and require stopping chemotherapy during treatment. Therefore, FOLFIRINOX is usually prescribed for patients ≤76 years old who have a good performance status (ECOG 0 or 1)^14^. To decrease the side effects and increase its tolerability, several institutions have used modified FOLFIRINOX. We conducted a systematic review and meta-analysis to assess the effectiveness and toxicities of modified FOLFIRINOX for patients with advanced pancreatic cancer compared to the conventional dosage.

## Methods

### Literature search

A systematic search was conducted to find eligible articles. Two investigators independently searched for prospective or retrospective studies (phase I-III trials, cohort studies, or case series) using Embase, PubMed, Web of Science, Scopus, and Cochrane without an upper-limit date until December 31, 2017. The search criteria included studies of advanced pancreatic cancer patients at any age who received any type of modified FOLFIRINOX in initial chemotherapy without language restrictions and no consideration of subsequent treatment. The preceding original regimen of FOLFIRINOX contained oxaliplatin 85 mg/m^2^, leucovorin 400 mg/m^2^, irinotecan 180 mg/m^2^, 5-fluorouracil (5-FU) bolus 400 mg/m^2^ and 5-fluorouracil (5-FU) 2400 mg/m^2^. Modified FOLFIRINOX was defined as at least one of the drugs was reduced and/or the removal of 5-FU bolus in FOLFIRINOX^14^.

The search strategy was as follows: ((‘folinic acid’/exp AND fluorouracil/exp AND irinotecan/exp AND oxaliplatin/exp AND ‘drug combination’/exp) or (Folfirinox):ab,ti) and (‘pancreas cancer’/de OR ‘pancreas tumor’/de OR ‘pancreas adenoma’/de OR ‘pancreas adenocarcinoma’/de OR ‘pancreas carcinoma’/de OR ‘pancreas islet cell carcinoma’/de OR (pancrea* NEAR/3 (cancer* OR neoplas* OR tumo* OR adenocarcinom* OR carcinom* OR adenom*)):ab,ti). For the detailed search strategy, see the supplement.

After removing duplicate articles, two investigators independently reviewed the abstracts. Studies were excluded if the study type was a review/meta-analysis, case report, comment, letter to the editor, or irrelevant literature. Differences between the investigators were resolved by a third-party investigator’s opinion. Full articles were then selected for further assessment, and articles with only abstracts were excluded. Other exclusion criteria included studies that used a regimen other than modified FOLFIRINOX, did not include the initial usage of modified FOLFIRINOX or dose adjusted by physician’s judgement without a specific time or presented the same patient cohort in another study. For details of the excluded articles, see the Supplement.

### Data extraction

General information was extracted from the foregoing selected publications and included the name of article, first authors, the name of journal, year of publication, study design, participating centres, country, observation sites, beginning and ending time, tumour stage, the composition of the modified FOLFIRINOX, its usage, number of patients, the ratio of males and females, average age, duration of follow-up, and performance status.

Survival was evaluated by the OS (6-month and 1-year) and PFS (6-month and 1-year) for the LAPC and MPC groups, which were extracted from the selected publications. If the survival rates were not directly available from the articles or authors, Engauge Digitizer was used to pool survival data from the Kaplan–Meier survival curve in each selected publication, especially for advanced pancreatic cancer reports for which the OS and PFS rates were not provided^18^. We chose the complete response (CR), partial response (PR), overall response (OR), stable disease (SD), disease control ratio (DCR), and progressive rate to evaluate the objective response to chemotherapy. The adverse events were calculated when they achieved grade III/IV. Grade III/IV toxicity includes neutropenia, febrile neutropenia, thrombocytopenia, anaemia, fatigue, vomiting, nausea, diarrhoea, neuropathy, and increased ALT.

### Statistical analysis

First, we used the Critical Appraisal Skill Program (CASP) to evaluate each study (supplement). The CASP is a critical appraisal tool that is used in observational studies to assess the methodological quality of the individual studies. Binary data were meta-analysed with the random-model generic inverse variance method. We used random-effects rather than fixed-effects models because of the heterogeneity in the initial treatment of advanced pancreatic cancer. We used the odds ratio as the effect measure method and then changed it to probability. The I^2^ statistics reflected the heterogeneity: I^2^ = 0% indicated no heterogeneity, I^2^ = (0%,25%) indicated low heterogeneity, I^2^ = [25%,50%) indicated mild heterogeneity, I^2^ = [50%,75%) indicated moderate heterogeneity, and I^2^ = [75%,100%] indicated high heterogeneity^19^. All analyses were performed in Review Manager version 5.3 and Excel 2010.

## Results

### Study search

**Figure 1:**
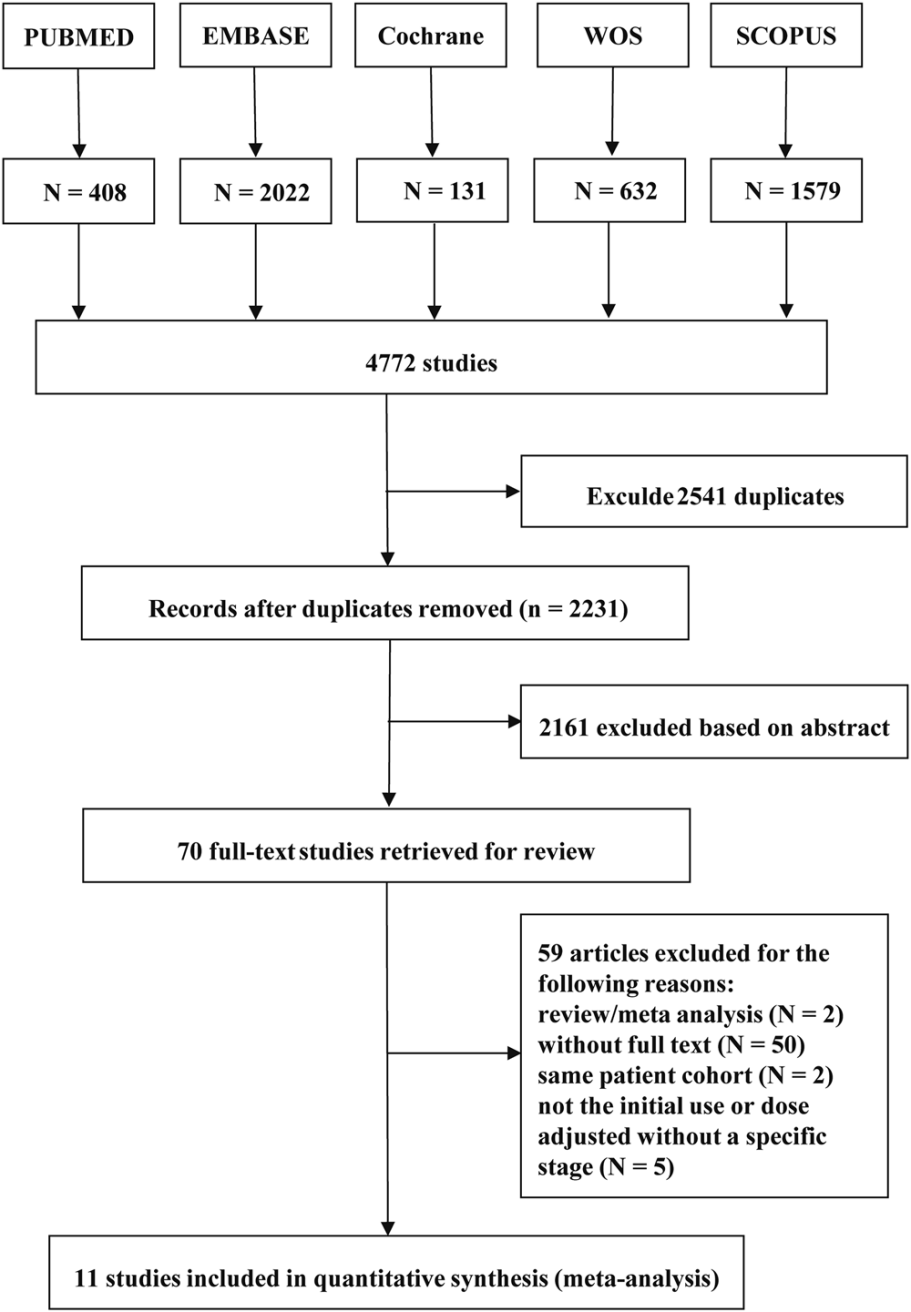
Flow chart for study search (PRISMA diagram).

Figure 1 is a flow diagram that shows the selection process for the searched studies. We searched all databases that are available. There were 4772 related studies identified from the initial literature search; 2541 studies were eliminated because of duplications. Only 70 studies were eligible upon abstract screening. After full-text screening, only 11 studies remained, and they were included in the final analysis^20–30^.

In these 11 studies, there were 563 patients, including 333 MPC and 230 LAPC. The number of patients who were treated with modified FOLFIRINOX ranged from 10 to 137. The average age in each study ranged from 60 to 65 years old (Table 1). Most patients’ performance status was 0 or 1, and a small portion had a score of 2^23^. Most of the studies removed the 5-FU bolus, but two studies reduced the dose from 400 mg/m^2^ to 300 mg/m^2 ^^27,29^. There was an overlap of population in one study^26,31^. The most usage of continuous infusion 5-FU was 2400 mg/m^2^, but one study increased it to 2800 mg/m^2^ or 3200 mg/m^2^ and eliminated the 5-FU bolus^28^. The most frequently used dose of oxaliplatin was the same as the normal FOLFIRINOX regimen, but two studies used 63.75 mg/m^2^ and 68 mg/m^2 ^^29,31^. The dosage of irinotecan ranged from 135 mg/m^2^ to 180 mg/m^2^. For the detailed modified FOLFIRINOX regimens, see Table 2.

**Table 1:** Summary of the included studies.

Author	Year of publication	Country	Start Time	End Time	Number of patients	Males (%)	PS (%) 0/1/2	Median age (range)	Tumour location Head/Neck	Tumour location Body/Tail	Number of LAPC	Number of MPC	Metastasis in liver	Metastasis in lungs	Metastasis in bones	Metastasis in peritoneal	Metastasis in lymph nodes
Stein^27^	2016	USA	2011.11	2014.1	68	62%	47/53/0	62(46–79)	44	18	31	37	20	12	0	14	15
Vivaldi^28^	2016	Italy	2008	2014	137	48%	67/33/0	60(33–75)	73	62	56	81	64	14	4	26	0
Mahaseth^21^	2013	USA	2010.6	2012.6	56	57%	22/76/2	63(36–78)	42	18	20	36	NA				
Ghorani^20^	2015	UK	2011.7	2014.5	18	44%	56/44/0	60(40–77)	10	1	3	15	NA				
Nanda^23^	2015	USA	2010.6	2013.3	29	41%	14/62/24	62(36–77)	24	5	29	0	NA				
Vočka^29^	2016	Czech	2013.1	2016.7	47	60%	57/43/0	62(40–72)	28	19	18	29	26	2	0	4	2
Liang*^26^	2016	China	2014.4	2015.1	76	67%	61/39/0	61(38–75)	NA	NA	14	62	49	1	0	7	10
Chllamma^25^	2016	Canada	2011.12	2014.7	66	NA	NA	64(28–76)	NA	NA	22	44	NA				
Takeda^24^	2015	Japan	2014.1	2015.7	10	40%	90/10/0	65(59–75)	4	6	2	8	NA				
Blazer^22^	2014	USA	2011.1	2013.8	25	48%	100%/0	62(40–81)	9	16	25	0	NA				
Yoshida^30^	2017	Japan	2014.1	2016.5	31	58%	81/19/0	64(49–72)	15	16	10	21	13	3	3	0	0
Total					563						230	333	172	32	7	51	27

*There was an overlap in their study population. PS: ECOG performance status.

**Table 2:** The detailed regimens of modified FOLFIRINOX.

Author	Chemotherapy regimens				
	Oxaliplatin	Folinic acid	Irinotecan	5-FU bolus	5-FU
Stein^27^	85 mg/m^2^	400 mg/m^2^	135 mg/m^2^	300 mg/m^2^	2400 mg/m^2^
Vivaldi^28^	85 mg/m^2^	200 mg/m^2^	150 mg/m^2^	None	2800 mg/m^2^
	85 mg/m^2^	200 mg/m^2^	165 mg/m^2^	None	3200 mg/m^2^
Mahaseth^21^	85 mg/m^2^	400 mg/m^2^	180 mg/m^2^	None	2400 mg/m^2^
Ghorani^20^	85 mg/m^2^	400 mg/m^2^	130–135 mg/m^2^	None	2400 mg/m^2^
Nanda^23^	85 mg/m^2^	400 mg/m^2^	180 mg/m^2^	None	2400 mg/m^2^
Vočka^29^	63.75 mg/m^2^	300 mg/m^2^	135 mg/m^2^	300	1800 mg/m^2^
Liang^26^	68 mg/m^2^	400 mg/m^2^	135 mg/m^2^	None	2400 mg/m^2^
Chllamma^25^	No specific regimen				
Takeda^24^	85 mg/m^2^	200 mg/m^2^	150 mg/m^2^	None	2400 mg/m^2^
Yoshida^30^	85 mg/m^2^	200 mg/m^2^	150 mg/m^2^	None	2400 mg/m^2^
Blazer^22^	85 mg/m^2^	None	165 mg/m^2^	None	2400 mg/m^2^

### Survival date

**Figure 2:**
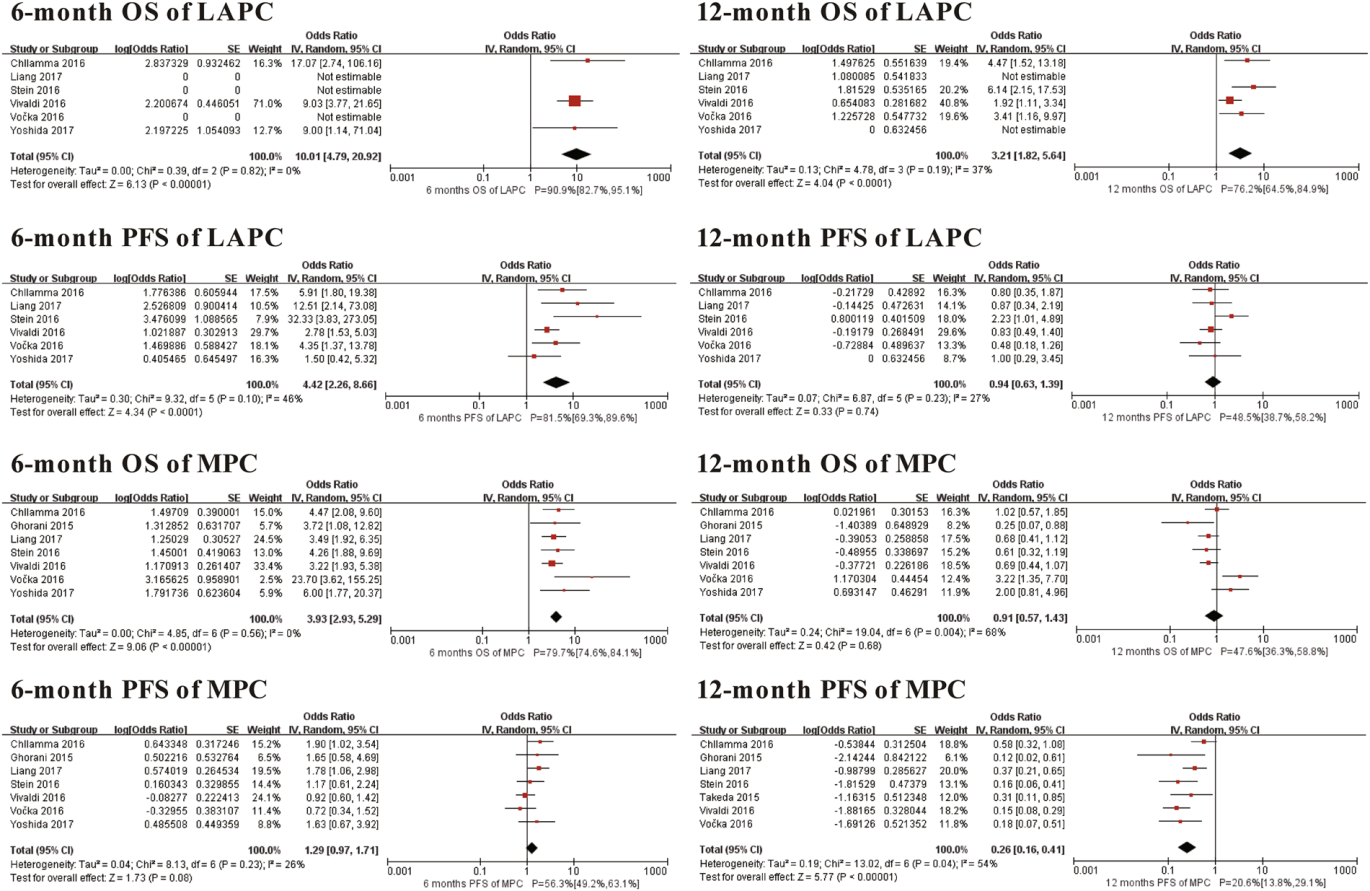
Meta-analysis for survival date. SE: standard error. IV: random-model generic inverse variance method. 95% CI: 95% confidence interval.

We divided advanced pancreatic cancer into LAPC and MPC to analyse the survival date because of the different prognoses between them. The pooled 6-month and 1-year OS rates of LAPC were 90.9 (95% CI 82.7–95.1%. I^2^ = 0%, *P* for Heterogeneity: 0.82) and 76.2% (95% CI 64.5–84.9%. I^2^ = 37%, *P* for Heterogeneity: 0.19). The pooled 6-month and 1-year PFS rates of LAPC were 81.5% (95% CI 69.3–89.6%. I^2^ = 46%, *P* for Heterogeneity: 0.10) and 48.5% (95% CI 38.7–58.2%. I^2^ = 27%, *P* for Heterogeneity: 0.23). The pooled 6-month and 1-year OS rates of MPC were 79.7% (95% CI 74.6–84.1%. I^2^ = 0%, *P* for Heterogeneity: 0.56) and 47.6% (95% CI 36.3–58.8%. I^2^ = 68%, *P* for Heterogeneity: 0.004). The pooled 6-month and 1-year PFS rates of MPC were 56.3% (95% CI 49.2–63.1%. I^2^ = 26%, *P* for Heterogeneity: 0.23) and 20.6% (95% CI 13.8–29.1%. I^2^ = 54%, *P* for Heterogeneity: 0.04) (Fig. 2).

### Response rates

**Figure 3:**
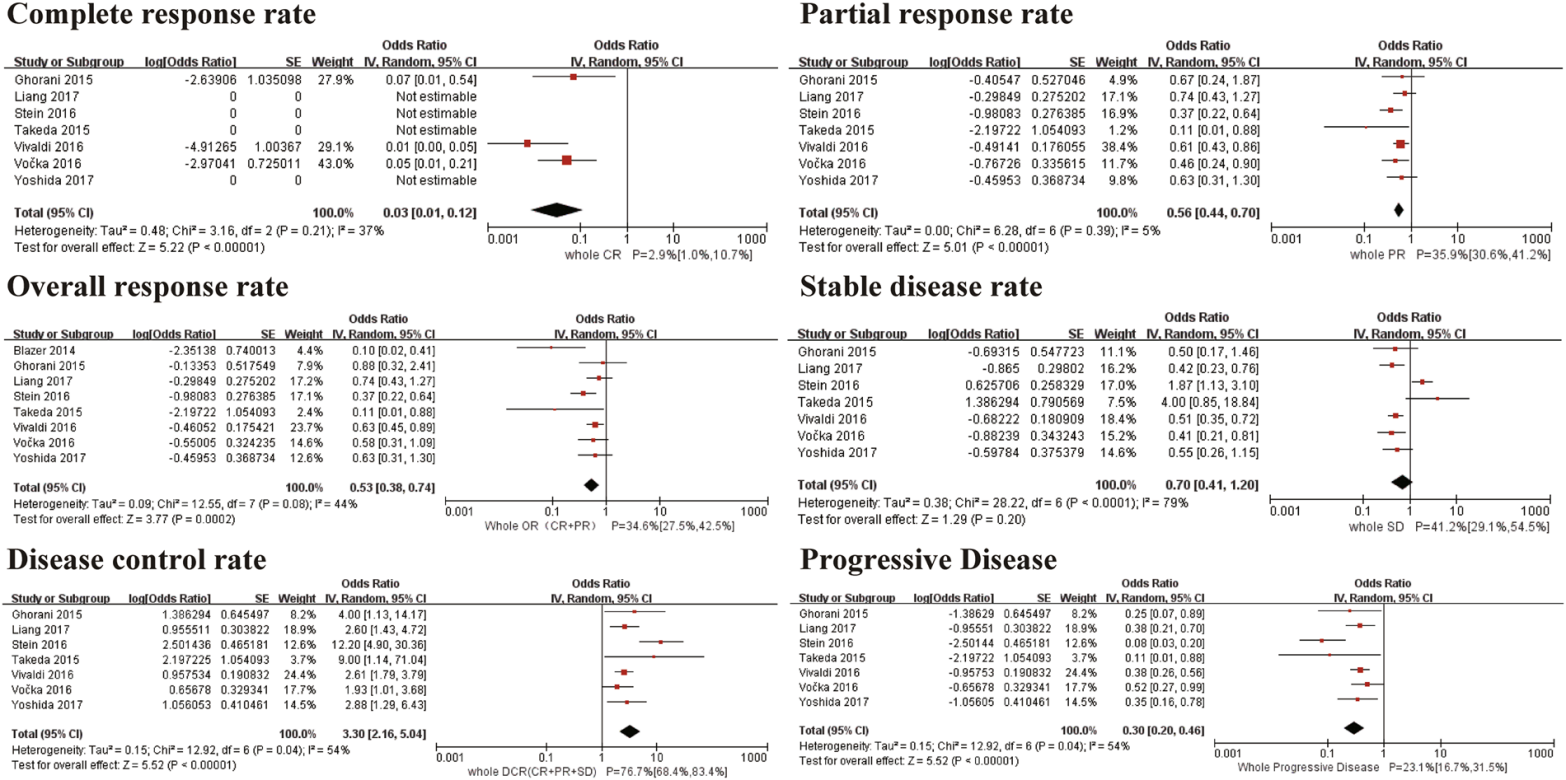
Meta-analysis for objective response rate. SE: standard error. IV: random-model generic inverse variance method. 95% CI: 95% confidence interval.

The pooled complete response rate (CR) was 2.9% (95% CI 1.0–10.7%. I^2^ = 37%, *P* for Heterogeneity: 0.21). The pooled partial response rate (PR) was 35.9% (95% CI 30.6–41.2%. I^2^ = 5%, *P* for Heterogeneity: 0.39). The pooled stable disease rate (SD) was 41.2% (95% CI 29.1–54.5%. I^2^ = 79%, *P* for Heterogeneity: <0.0001). The pooled overall response rate (OR) was 34.6% (95% CI 27.5–42.5%. I^2^ = 44%, *P* for Heterogeneity: 0.08). The pooled disease control rate (DCR) was 76.7% (95% CI 68.4–83.4%. I^2^ = 54%, *P* for Heterogeneity: 0.04). The pooled progressive disease was 23.1% (95% CI 16.7–31.5%. I^2^ = 54%, *P* for Heterogeneity: 0.04) (Fig. 3 and Table 3).

**Table 3:** The chemotherapy response to modified FOLFIRINOX.

Author	CR	PR	OR	SD	DCR	Number of patients
Stein^27^	0	18	18	43	61	66
Vivaldi^28^	1	52	53	46	99	137
Ghorani^20^	1	6	7	5	12	15
Vočka^29^	2	13	15	12	27	41
Liang^26^	0	23	23	16	39	54
Takeda^24^	0	1	1	8	9	10
Blazer^22^	NA	NA	2	NA	NA	23
Yoshida^30^	0	12	12	11	23	31
Total	4	125	131	141	270	377

CR: complete response rate. PR: partial response rate. SD: stable disease rate. OR: overall response rate. DCR: disease control rate.

### Adverse events

**Figure 4:**
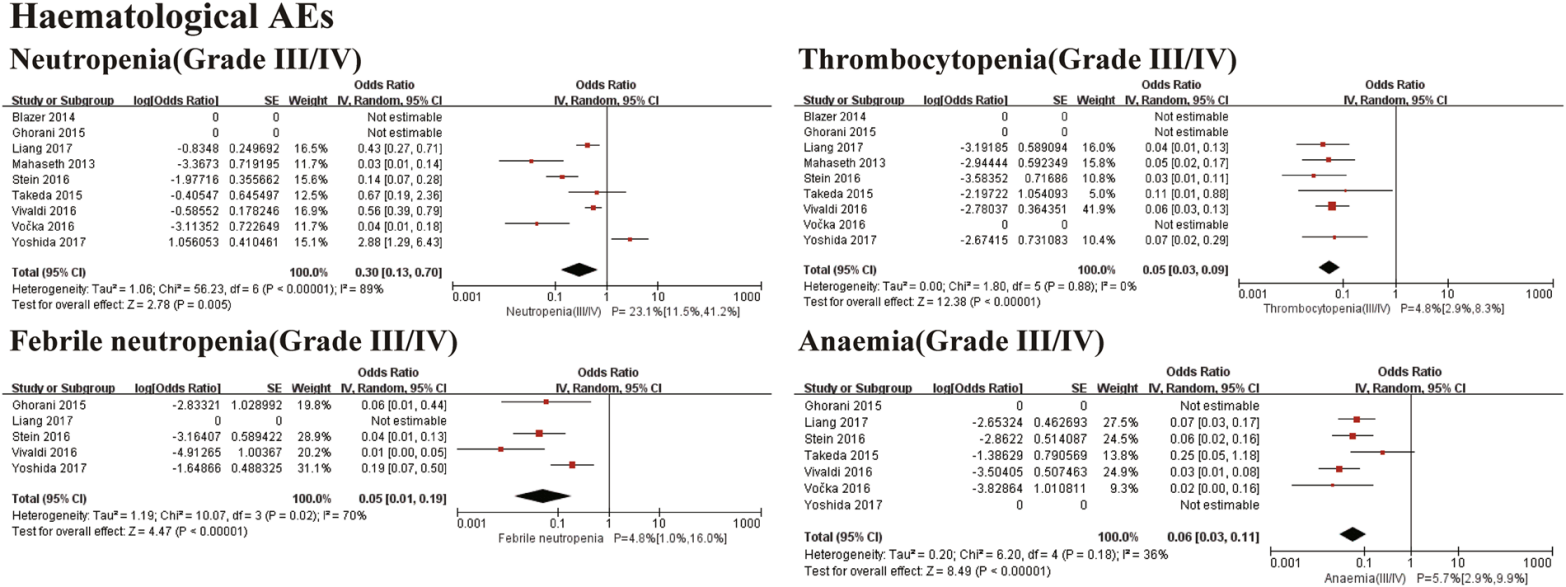
Meta-analysis for adverse events of haematological AEs. SE: standard error. IV: random-model generic inverse variance method. 95% CI: 95% confidence interval.

There were 344 grade III/IV adverse events in our study (Table 4). Figure 4 shows the pooled event rates for grade III/IV adverse events. The pooled grade III/IV incidences of neutropenia, febrile neutropenia, thrombocytopenia, and anaemia were 23.1% (95% CI 11.5–41.2%. I^2^ = 89%, *P* for Heterogeneity: <0.00001), 4.8% (95% CI 1.0–16%. I^2^ = 70%, *P* for Heterogeneity: 0.02), 4.8% (95% CI 2.9–8.3%. I^2^ = 0%, *P* for Heterogeneity: 0.88), and 5.7% (95% CI 2.9–9.9%. I^2^ = 36%, *P* for Heterogeneity: 0.18).

**Table 4:** The adverse events of modified FOLFIRINOX.

Adverse events	Stein	Vivaldi	Mahaseth	Ghorani	Nanda	Vočka	Chllamma	Takeda	Yoshida	Liang	Blazer	Total patients
Neutropenia	9	49	2	0	NA	2	NA	4	26	23	0	115
Thrombocytopenia	2	8	3	0		0		1	2	3	0	19
Febrile neutropenia	3	1	NA	1		NA		NA	5	0	NA	10
Anaemia	4	4	NA	0		1		2	0	5	NA	16
Fatigue	9	NA	8	1		NA		NA	NA	0	4	22
Nausea	NA	10	NA	4		4		2	1	NA	2	23
Diarrhoea	12	11	8	3		4		0	2	1	6	47
Vomiting	2	5	5	5		3		0	1	1	0	22
Neuropathy	2	3	3	0		1		NA	3	0	0	12
Increased ALT	3	6	NA	0		1		NA	NA	9	NA	19
Asthenia	NA	2	NA	NA		NA		NA	NA	NA	NA	2
Thromboembolic event	3	6	0	0		NA		NA	NA	0	NA	9
Stomatitis	NA	9	NA	NA		NA		NA	NA	NA	NA	9
Gastrointestinal haemorrhage	NA	NA	NA	NA		NA		1	NA	NA	NA	1
Anorexia	NA	4	NA	NA		NA		0	2	NA	NA	6
Allergic reaction	NA	NA	2	NA		NA		NA	NA	NA	NA	2
Mucositis	NA	NA	1	NA		NA		NA	0	NA	0	1
Infection	NA	NA	3	NA		NA		NA	NA	3	NA	6
Dysarthria	NA	NA	NA	1		NA		NA	NA	NA	NA	1
Hyperbilirubinemia	NA	NA	NA	NA		NA		NA	NA	NA	2	2
Total events	49	118	35	15	NA	16	NA	10	42	45	14	344

**Figure 5:**
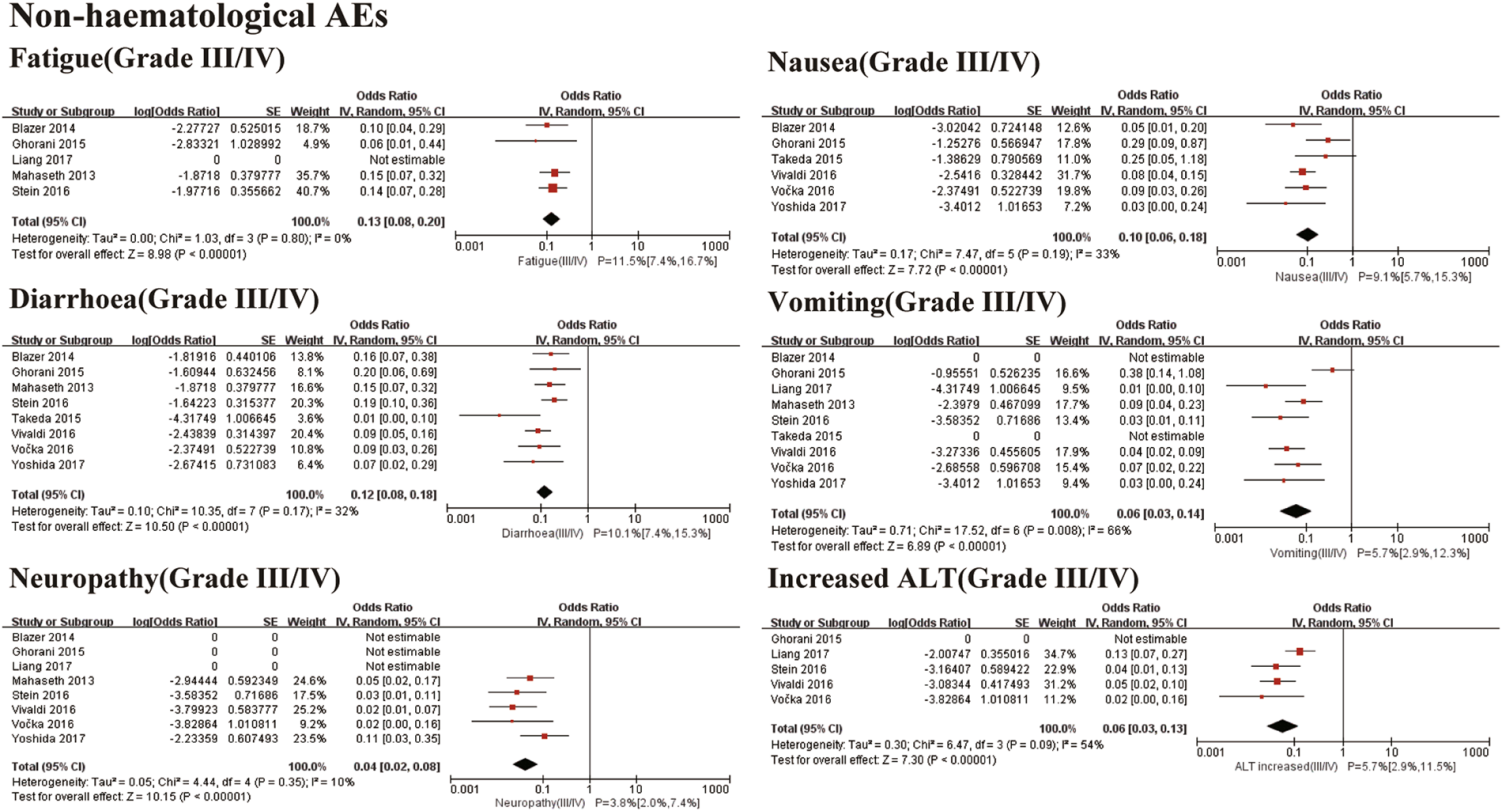
Meta-analysis for adverse events of non-haematological AEs. SE: standard error. IV: random-model generic inverse variance method. 95% CI: 95% confidence interval.

The pooled incidences of non-haematological AEs were as follows: fatigue 11.5% (95% CI 7.4–16.7%. I^2^ = 0%, *P* for Heterogeneity: 0.80), nausea 9.1% (95% CI 5.7–15.3%. I^2^ = 33%, *P* for Heterogeneity: 0.19), diarrhoea 10.1% (95% CI 7.4–15.3%. I^2^ = 32%, *P* for Heterogeneity: 0.17), vomiting 5.7% (95% CI 2.9–12.3%. I^2^ = 66%, *P* for Heterogeneity: 0.008), neuropathy 3.8% (95% CI 2.0–7.4%. I^2^ = 10%, *P* for Heterogeneity: 0.35), and increased ALT 5.7% (95% CI 2.9–11.5%. I^2^ = 54%, *P* for Heterogeneity: 0.09) (Fig. 5).

## Discussion

Our systematic review and meta-analysis considered 11 studies, which contained 563 patients with advanced pancreatic cancer treated with modified FOLFIRINOX. Previously, FOLFIRINOX was used to treat advanced pancreatic adenocarcinoma and demonstrated a better therapeutic benefit than gemcitabine (GEM)^32^. Although the dosage of FOLFIRINOX was reduced, the 12-month survival rate was still much higher than those of gemcitabine and its combinational regimen, with the first at 76.2% in LAPC and 47.6% in MPC, compared to 18–37.2%^11,33–36^. Since then, many clinical studies have been assessed the treatment of advanced pancreatic cancer by using modified FOLFIRINOX. Compared to the preceding original regimen of FOLFIRINOX, the OS and PFS at 6 and 12 months for modified FOLFIRINOX were nearly equivalent^14,20,37,38^. Similar to the data obtained for OS and PFS, as mentioned above, the response rate of modified FOLFIRINOX was also comparable to that of the original regimen^14,20,37,38^. Nevertheless, the favourable overall survival after modified FOLFIRINOX might be partly attributable to patient selection from many non-randomized studies.

For the adverse events, the pooled rates of grade III/IV adverse events were lower than those of the FOLFIRINOX group; some were even lower than the GEM group^14,39,40^, such as anaemia, fatigue and vomiting. Concomitantly, a prospective phase II study of dose-attenuated treatment found that modified FOLFIRINOX could significantly reduce the occurrence of vomiting and fatigue^41^. As we know, in practice, when patients experience serious adverse events during continuous FOLFIRINOX chemotherapy, the strategy for physicians is to reduce the dosage or even stop the chemotherapy. Therefore, modified FOLFIRINOX is a good choice at the beginning of therapy, particularly for those with poor performance status. Modified FOLFIRINOX provides a relatively mild intervention and thus induces lower adverse events, thereby ensuring the continuity of chemotherapy. Interestingly, there was a great difference between the Asian group and Euromerican group in neutropenia (48.5% [20.6%, 77.4%] vs 10.7% [2.9%, 31.3%]). This may be due to different genetic traits between the ethnic groups.

In general, the modified FOLFIRINOX regimen could provide good survival benefits for patients with advanced pancreatic cancer by increasing the OS and PFS and causing fewer adverse events. Our findings suggest that the dosage attenuation of initial FOLFIRINOX improves its tolerability without compromising its efficacy. Compared to the original regimen of FOLFIRINOX, modified FOLFIRINOX may be more applicable for patients with poor performance status. However, there were multiple combinations of the four drugs in which the 5-FU bolus was removed; which combination is the best for different ethnic groups or different healthy conditions remains a significant question. Clinical trials are still needed to justify the best combination for modified FOLFIRINOX. At last, although most of the studies that we chose were non-randomized and some even had a retrospective design that might bring bias, the current meta-analysis could provide constructive information for clinicians and patients.

## Electronic supplementary material

Supplementary Dataset
