# Supplementary material for: The benefits of modified FOLFIRINOX for advanced pancreatic cancer and its induced adverse events: a systematic review and meta-analysis
Source: Sci Rep. 2018 Jun 6;8:8666. doi: 10.1038/s41598-018-26811-9 (PMC5989209; doi:10.1038/s41598-018-26811-9)
Supplement: Supplementary file 1 — Supplementary Dataset [file 41598_2018_26811_MOESM1_ESM.pdf]

Supplementary appendix for: The benefits of modified FOLFIRINOX for advanced pancreatic cancer and its induced adverse events: a systematic review and meta analysis

Author:Hongxuan Tong, Zhu Fan, Biyuan, Liu Tao Lu

Detailed search strategy

Pubmed:

#1 ("Pancreatic Neoplasms"[Mesh]) OR (Neoplasm, Pancreatic) OR (Pancreatic Neoplasm) OR (Pancreas Neoplasms) OR (Neoplasm, Pancreas) OR (Neoplasms, Pancreas) OR (Pancreas Neoplasm) OR (Neoplasms, Pancreatic) OR (Cancer of Pancreas) OR (Pancreas Cancers) OR (Pancreas Cancer) OR (Cancer, Pancreas) OR (Cancers, Pancreas) OR (Pancreatic Cancer) OR (Cancer, Pancreatic) OR (Cancers, Pancreatic) OR (Pancreatic Cancers) OR (Cancer of the Pancreas)

#2 ("Fluorouracil"[Mesh]) OR (5-FU) OR (Fluoruracil) OR (5-Fluorouracil) OR (5 Fluorouracil) OR (5FU) OR (5-FU Lederle) OR (5 FU Lederle) OR (Riemser Brand of Fluorouracil) OR (5-FU medac) OR (5 FU medac) OR (medac Brand of Fluorouracil) OR (5-HU Hexal) OR (5 HU Hexal) OR (Hexal Brand of Fluorouracil) OR (Adrucil) OR (Teva Brand of Fluorouracil) OR (Fluorouracil Teva Brand) OR (Carac) OR (Dermik Brand of Fluorouracil) OR (Efudix) OR (Dermatech Brand of Fluorouracil) OR (Roche Brand of Fluorouracil) OR (Fluoro-Uracile ICN) OR (Fluoro Uracile ICN) OR (ICN Brand of Fluorouracil) OR (CSP Brand of Fluorouracil) OR (Efudex) OR (Fluoroplex) OR (Flurodex) OR (Allergan Brand of Fluorouracil) OR (Fluorouracil Mononitrate) OR (Fluorouracil Monopotassium Salt) OR (Fluorouracil Monosodium Salt) OR (Fluorouracil Potassium Salt) OR (Fluorouracil-GRY) OR (Fluorouracil GRY) OR (Gry Brand of Fluorouracil) OR (Fluorouracile Dakota) OR (Dakota, Fluorouracile) OR (Dakota Brand of Fluorouracil) OR (Fluorouracilo Ferrer Far) OR (Ferrer Brand of Fluorouracil) OR (Fluracedyl) OR (Pharmachemie Brand of Fluorouracil Monosodium Salt) OR (Haemato-fu) OR (Haemato fu) OR (Haemato Brand of Fluorouracil) OR (Neofluor) OR (Neocorp Brand of Fluorouracil) OR (Onkofluor) OR (Onkoworks Brand of Fluorouracil) OR (Ribofluor) OR (ribosepharm Brand of Fluorouracil) OR (5-Fluorouracil-biosyn) OR (5 Fluorouracil biosyn) OR (biosyn Brand of Fluorouracil)

#3 ("oxaliplatin" [Supplementary Concept]) OR (1,2-diamminocyclohexane(trans-1)oxolatoplatinum(II)) OR (oxalato-(1,2-cyclohexanediamine)platinum II) OR (L-OHP cpd) OR (oxaliplatine) OR (1,2-diaminocyclohexane platinum oxalate) OR (platinum(II)-1,2-cyclohexanediamine oxalate) OR (cis-oxalato-(trans-l)-1,2-diaminocyclohexane-platinum(II)) OR (oxaliplatin, (SP-4-3-(cis))-isomer) OR (oxaliplatin, (SP-4-2-(1R-trans))-isomer) OR (oxaliplatin, (SP-4-2-(1S-trans))-isomer) OR (ACT 078) OR (ACT-078) OR (Eloxatine) OR (Sanofi Synthelabo brand of oxaliplatin) OR (Sanofi brand of oxaliplatin) OR (Eloxatin)

#4 ("Leucovorin"[Mesh]) OR (Leukovorum) OR (Leukovorin) OR (Folinic Acid) OR (Acid, Folinic) OR (Folinic Acid-SF) OR (Folinic Acid SF) OR (Leucovorin, (D)-Isomer) OR (Leucovorin, (DL)-Isomer) OR (Leucovorin, (R)-Isomer) OR (Leucovorin, Calcium (1:1) Salt) OR (Leucovorin, Calcium (1:1) Salt, Pentahydrate) OR (Leucovorin, Monosodium Salt) OR (Monosodium Salt Leucovorin) OR (N(5)-Formyltetrahydrofolate) OR

(5-Formyltetrahydropteroylglutamate) OR (5 Formyltetrahydropteroylglutamate) OR (5-Formyltetrahydrofolate) OR (5 Formyltetrahydrofolate) OR (Wellcovorin) OR (Citrovorum Factor) OR (Factor, Citrovorum) OR (Calcium Leucovorin) OR (Leucovorin, Calcium) OR (Calcium Folinate) OR (Folinate, Calcium) OR (Leucovorin, Calcium (1:1) Salt, (DL)-Isomer)
#5 ("irinotecan" [Supplementary Concept]) OR (Irrinotecan) OR (Camptosar) OR (SN 38 11) OR (SN-38-11) OR (NK012 compound) OR (SN 38) OR (SN38 cpd) OR (SN-38) OR (7-ethyl-10-hydroxycamptothecin) OR (irinotecan hydrochloride) OR (camptothecin-11) OR (CPT 11) OR (CPT-11)
#6 FOLFIRINOX
#7 #2 and #3 and #4 and #5
#8 #6 or #7
#9 #8 and #1

Conchrane:
#1 (Pancreatic Neoplasms) or (Neoplasm, Pancreatic) or (Pancreatic Neoplasm) or (Pancreas Neoplasms) or (Neoplasm, Pancreas) or (Neoplasms, Pancreas) or (Pancreas Neoplasm) or (Neoplasms, Pancreatic) or (Cancer of Pancreas) or (Pancreas Cancers) or (Pancreas Cancer) or (Cancer, Pancreas) or (Cancers, Pancreas) or (Pancreatic Cancer) or (Cancer, Pancreatic) or (Cancers, Pancreatic) or (Pancreatic Cancers) or (Cancer of the Pancreas)
#2 (FOLFIRINOX)
#3 (Fluorouracil) or (5-FU) or (Fluoruracil) or (5-Fluorouracil) or (5 Fluorouracil) or (5FU) or (5-FU Lederle) or (5 FU Lederle) or (Riemser Brand of Fluorouracil) or (5-FU medac) or (5 FU medac) or (medac Brand of Fluorouracil) or (5-HU Hexal) or (5 HU Hexal) or (Hexal Brand of Fluorouracil) or (Adrucil) or (Teva Brand of Fluorouracil) or (Fluorouracil Teva Brand) or (Carac) or (Dermik Brand of Fluorouracil) or (Efudix) or (Dermatech Brand of Fluorouracil) or (Roche Brand of Fluorouracil) or (Fluoro-Uracile ICN) or (Fluoro Uracile ICN) or (ICN Brand of Fluorouracil) or (CSP Brand of Fluorouracil) or (Efudex) or (Fluoroplex) or (Flurodex) or (Allergan Brand of Fluorouracil) or (Fluorouracil Mononitrate) or (Fluorouracil Monopotassium Salt) or (Fluorouracil Monosodium Salt) or (Fluorouracil Potassium Salt) or (Fluorouracil-GRY) or (Fluorouracil GRY) or (Gry Brand of Fluorouracil) or (Fluorouracile Dakota) or (Dakota, Fluorouracile) or (Dakota Brand of Fluorouracil) or (Fluorouracilo Ferrer Far) or (Ferrer Brand of Fluorouracil) or (Fluracedyl) or (Pharmachemie Brand of Fluorouracil Monosodium Salt) or (Haemato-fu) or (Haemato fu) or (Haemato Brand of Fluorouracil) or (Neofluor) or (Neocorp Brand of Fluorouracil) or (Onkofluor) or (Onkoworks Brand of Fluorouracil) or (Ribofluor) or (ribosepharm Brand of Fluorouracil) or (5-Fluorouracil-biosyn) or (5 Fluorouracil biosyn) or (biosyn Brand of Fluorouracil)
#4 ((oxaliplatin) or (1,2-diamminocyclohexane (trans-1) oxolatoplatinum (II)) or (oxalate (1,2-cyclohexanediamine) platinum ii) or (l-ohp cpd) or (oxaliplatin) or (1,2-diaminocyclohexane platinum oxalate) or (platinum (II) -1,2-cyclohexanediamine oxalate) or (bis oxalato (trans-l) -1,2-diaminocyclohexane-platinum (II)) or (oxaliplatin, (SP-4-3- (cis)) isomer) or (oxaliplatin, (SP-4-2- (1R-trans)) isomer) or (oxaliplatin, (SP-4-2- (1S-trans)) isomer) or (ACT 078) or ACT-078) or (eloxatin) or (sanofi synthelabo brand of oxaliplatin) or (sanofi brand of oxaliplatin) or eloxatin
#5 (leucovorin) or (leukovorum) or (leukovorin) or (folinic acid) or (acid, folinic) or (folinic

acid-sf) or (folinic acid sf) or (leucovorin, (D) isomer) or (leucovorin, (DL) isomer) or (leucovorin, (R) isomer) or (leucovorin, calcium 1 1 salt) or (leucovorin, calcium 1 1 salt, pentahydrate) or (leucovorin, monosodium salt) or (monosodium salt leucovorin) or (N (5) formyltetrahydrofolate) or (5-Formyltetrahydropteroylglutamate) or (5 formyltetrahydropteroylglutamate) or (5-Formyltetrahydrofolate) or (5 formyltetrahydrofolate) or (wellcovorin) or (citrovorum factor) or (factor, citrovorum) or (calcium leucovorin) or (leucovorin, calcium) or (calcium folinate) or (folinate, calcium) or (leucovorin, calcium 1 1 salt, (DL) isomer)

#6 (irinotecan) or irinotecan or (camptosar) or (SN 38 11) or (SN-38-11) or (nk012 compound) or (SN 38) or (SN38 cpd) or (SN-38) or (7-ethyl-10-hydroxycamptothecin) or (irinotecan hydrochloride) or (camptothecin-11) or (CPT 11) or (CPT-11)

#7 #3 and #4 and #5 and #6

#8 #7 or #2

#9 #1 and #8

Embase:

(('folinic acid'/exp AND fluorouracil/exp AND irinotecan/exp AND oxaliplatin/exp AND 'drug combination'/exp) or (Folfirinox):ab,ti) and ('pancreas cancer'/de OR 'pancreas tumor'/de OR 'pancreas adenoma'/de OR 'pancreas adenocarcinoma'/de OR 'pancreas carcinoma'/de OR 'pancreas islet cell carcinoma'/de OR (pancrea* NEAR/3 (cancer* OR neoplas* OR tumo* OR adenocarcinom* OR carcinom* OR adenom*)):ab,ti)

Scopus:

(((TITLE-ABS-KEY((Fluor*uracil*) OR (5*FU) OR (5*HU Hexal) OR (Adrucil) OR (Carac) OR (Efudix) OR (Efudex) OR (Fluoroplex) OR (Flurodex) OR (Fluracedyl) OR (Haemato*fu) OR (Neofluor) OR (Onkofluor) OR (Ribofluor))) and (TITLE-ABS-KEY((oxaliplatin) OR (L-OHP cpd) OR (oxaliplatine) OR (1,2-diaminocyclohexane platinum oxalate) OR (ACT*078) OR (Eloxatine) OR (Eloxatin))) and (TITLE-ABS-KEY((Leu*ovorin) OR (Leukovorum) OR (Folinic Acid) OR (Wellcovorin) OR (Citrovorum Factor) OR (Calcium Folinate) OR (Formyltetrahydro*))) and (TITLE-ABS-KEY((ir*inotecan) OR (Camptosar) OR (NK012 compound) OR (SN*38) OR (7-ethyl-10-hydroxycamptothecin) OR (camptothecin-11) OR (CPT*11)))) or (TITLE-ABS-KEY(Folfirinox))) and (TITLE-ABS-KEY(pancrea* AND (cancer* OR neoplas* OR tumo* OR adenocarcinom* OR carcinom* OR adenom*)))

WEB OF SCIENCE:

1 TS=(Folfirinox)

2 TS=(pancrea* AND (cancer* OR neoplas* OR tumo* OR adenocarcinom* OR carcinom* OR adenom*))

3 TS=((Fluor*uracil*) OR (5*FU) OR (5*HU Hexal) OR (Adrucil) OR (Carac) OR (Efudix) OR (Efudex) OR (Fluoroplex) OR (Flurodex) OR (Fluracedyl) OR (Haemato*fu) OR (Neofluor) OR (Onkofluor) OR (Ribofluor))

4 TS=((oxaliplatin) OR (L-OHP cpd) OR (oxaliplatine) OR (1,2-diaminocyclohexane platinum oxalate) OR (ACT*078) OR (Eloxatine) OR (Eloxatin))

5 TS=((Leu*ovorin) OR (Leukovorum) OR (Folinic Acid) OR (Wellcovorin) OR (Citrovorum Factor) OR (Calcium Folinate) OR (Formyltetrahydro*))

6 TS=((ir*inotecan) OR (Camptosar) OR (NK012 compound) OR (SN*38) OR

(7-ethyl-10-hydroxycamptothecin) OR (camptothecin-11) OR (CPT*11))
7 #6 AND #5 AND #4 AND #3
8 #7 OR #1
9 #8 AND #2

Excluded studies after full text assessment

number	Excluded studies	Reason
1	Modified folfirinox regimen for advanced pancreatic cancer: A single center's experience from China	same patient cohort and without full text
2	Safety and efficacy of modified dose-attenuated FOLFIRINOX chemotherapy in patients over 65 years with advanced pancreatic adenocarcinoma	without full text
3	Safety and efficacy of modified dose-attenuated FOLFIRINOX chemotherapy in patients over 65 years with advanced pancreatic adenocarcinoma	without full text
4	Folfirinage: Tolerance and efficacy of folfirinox in elderly patients with advanced pancreatic adenocarcinoma	without full text
5	Resection of locally advanced pancreatic cancer after neoadjuvant chemotherapy with modified folfirinox: A prospective phase II study	without full text
6	Tolerability and efficacy of modified FOLFIRINOX (mFOLFIRINOX) in patients with borderline-resectable pancreatic cancer (BRPC) and locally advanced unresectable pancreatic cancer (LAURPC)	without full text
7	Neoadjuvant FOLFIRINOX combined with aggressive surgical resection allows potentially curative therapy for borderline resectable and locally advanced pancreatic cancer	without full text
8	Compa rative analysis of metabolic response to clinical outcome in metastatic pancreatic adenocarcinoma following modified dose folfirinox	without full text
9	The impact of folfirinox chemotherapy on the treatment pattern of patients with pancreas cancer seen at a tertiary referral centre in the UK	without full text
10	The role of the FOLFIRINOX regimen for advanced pancreatic cancer	without full text
11	Retrospective research for efficacy and safety of modified FOLFIRINOX as 1st line therapy for advanced pancreatic cancer	a review
12	Folfirinox in pancreatic cancer: The national cancer institute of Milan single experience	without full text
13	Folfirinox in advanced pancreatic cancer: A single-center experience	without full text
14	Folfirinox in locally advanced pancreatic cancer: The massachusetts general hospital cancer center experience	without full text
15	Modified folfoxiri in advanced pancreatic cancer	not the initial use or does adjusted without a specific stage
16	Experience with FOLFIRINOX regimen in patients over 65 years old diagnosed with pancreatic adenocarcinoma: Efficacy and safety data	without full text
17	Multi-institutional experience using 5-fluorouracil, leucovorin, irinotecan, and oxaliplatin (FOLFIRINOX) in patients with pancreatic cancer (PCA)	without full text
18	Single-institution experience with FOLFIRINOX in advanced pancreatic cancer (PC)	without full text
19	Single institution experience with FOLFIRINOX in advanced pancreatic cancer (PC)	without full text
20	Folfirinox in pancreatic cancer-experience with a novel scheme of induction, maintenance, treatment pause and re-induction	without full text
21	FOLFIRINOX for locally advanced and metastatic pancreatic ductal adenocarcinoma	without full text

22	FOLFIRINOX with modified regimen therapy for advanced pancreatic cancer in Okayama university hospital	without full text
23	FOLFIRINOX for the treatment of advanced pancreatic cancer: U.K. West Midlands experience	without full text
24	Final analysis of a phase II study of Yale-modified FOLFIRINOX (mFOLFIRINOX) in metastatic pancreatic cancer (MPC)	without full text
25	Interim analysis of a phase II study of dose-modified FOLFIRINOX (mFOLFIRINOX) in locally advanced (LAPC) and metastatic pancreatic cancer (MPC)	without full text
26	Full dose neoadjuvant FOLFIRINOX is associated with prolonged survival in patients with locally advanced pancreatic adenocarcinoma	without full text
27	Retrospective study of FOLFIRINOX in early intolerable patients with unresectable pancreatic cancer in our institution	not the initial use or does adjusted without a specific stage
28	Does reduced dose of FOLFIRINOX guarantee tumor response in unresectable pancreatic cancer? suggestion for the minimal relative dose intensity	without full text
29	Activity of front-line FOLFIRINOX (FFX) in stage III/IV pancreatic adenocarcinoma (PC) at Memorial Sloan-Kettering Cancer Center (MSKCC)	without full text
30	Safety and efficacy of modified FOLFIRINOX in pancreatic cancer: A retrospective experience	without full text
31	A registry of real-world clinical practice on the use of FOLFIRINOX (FFX) in advanced pancreatic cancer (aPC) patients in Canada	without full text
32	Investigation of the tolerability of FOLFIRINOX in patients with unresectable advanced pancreatic cancer: Single-institution experience in Japan	without full text
33	FOLFIRINOX: From the ACCORD study to 2014	without full text
34	A single institution retrospective review of efficacy, toxicity, and symptom burden in patients (pts) with pancreatic cancer (PaC) receiving FOLFIRINOX chemotherapy	a review
35	Multi-institutional experience with FOLFIRINOX in pancreatic adenocarcinoma	without full text
36	Washington University experience with FOLFIRINOX in pancreatic cancer	not the initial use or does adjusted without a specific stage
37	FOLFIRINOX in Locally Advanced and Metastatic Pancreatic Cancer: A Single Centre Cohort Study	without full text
38	Safety profile of modified FOLFIRINOX	not the initial use or does adjusted without a specific stage
39	A UGT1A1 genotype-guided dosing study of modified FOLFIRINOX (mFOLFIRINOX) in previously untreated patients (pts) with advanced gastrointestinal malignancies	without full text
40	Phase II study of Yale modified FOLFIRINOX (mFOLFIRINOX) in locally advanced pancreatic cancer (LAPC)	without full text
41	Phase II study of modified FOLFIRINOX for chemotherapynaive patients with metastatic pancreatic cancer	without full text
42	Effects of dose-modified FOLFIRINOX on toxicity and effectiveness in Japanese patients with unresectable pancreatic cancer (PC)	without full text
43	Safety and efficacy of dose-modified FOLFIRINOX on toxicity and effectiveness in Japanese patients with unresectable pancreatic cancer (PC)	without full text
44	Modified FOLFIRINOX in pancreatic cancer patients with double variant type of UGT1A1 polymorphism	without full text
45	First-line treatment with folfirinox in advanced, inoperable pancreatic cancer (APDAC) patients (PTS): Supportive measures optimization for a safe administration in routine clinical practice	without full text
46	Optimizing supportive measures for the safe administration of FOLFIRINOX as first-line treatment in	without full text

	advanced, inoperable pancreatic cancer (aPDAC) patients (pts) in routine clinical practice	
47	FOLFIRNOX for advanced pancreatic cancer (aPDAC) according to schedule and dose modifications: Clinical feasibility and impact of supportive care	without full text
48	Neoadjuvant modified folfoxiri in locally advanced pancreatic cancer	without full text
49	Phase II study of neoadjuvant chemotherapy with modified FOLFOXIRI in borderline resectable or unresectable stage III pancreatic cancer	without full text
50	Prognostic model for patients (PTS) with advanced pancreatic cancer (aPC) receiving first-line modified FOLFIRINOX (mFOLFIRINOX)	without full text
51	Modified FOLFIRINOX as initial treatment of advanced pancreatic cancer: A single cancer center experience	without full text
52	Safety and efficacy of modified FOLFIRINOX in patients with metastatic pancreatic cancer	without full text
53	Randomized phase II study of modified FOLFIRINOX versus gemcitabine plus nab-paclitaxel combination therapy for locally advanced pancreatic cancer: Japan Clinical Oncology Group Study	without full text
54	The role of modified FOLFIRINOX regimen in unresectable advanced pancreatic cancer: A retrospective clinical study	without full text
55	Analysis of DPYD and UGT1A1 genotype in patients with advanced pancreatic cancer treated with modified FOLFIRINOX	without full text
56	Clinical outcome of modified FOLFIRINOX versus Gemcitabine plus nab-paclitaxel as first line chemotherapy in metastatic pancreatic cancer	without full text
57	Phase 2 study of 1st-line modified FOLFIRINOX in unresectable advanced pancreatic cancer: Multicenter prospective study	without full text
58	The impact of UGT1A1 genetic polymorphism on safety in unresectable pancreatic cancer patients receiving FOLFIRINOX therapy: A subset analysis of JASPAC 06 study	without full text
59	Modified FOLFIRINOX for advanced pancreatic cancer: a tertiary center experience from China	same patient cohort

Table 1. Quality assessment according to Critical Appraisal Skill Program.

**CASP factors**	**Stein**	**Vivaldi**	**Takeda**	**Mahaseth**	**Ghorani**	**Nanda**	**Vočka**	**Li**	**Chllamma**	**Blazer**	**Yoshida**
study type	single arm	prospective cohort studies	retrospective cohort studies	retrospective cohort studies	retrospective cohort studies	retrospective cohort studies	retrospective cohort studies	prospective cohort studies	retrospective cohort studies	retrospective cohort studies	single arm
Clearly focused issue	YES	YES	YES	YES	YES	YES	YES	YES	YES	YES	YES
Acceptable recruitment	YES	YES	YES	YES	YES	YES	YES	YES	YES	YES	YES
Exposure minimise bias	YES	YES	YES	YES	YES	YES	YES	YES	YES	YES	can't tell

Outcome minimise bias	YES	YES	YES	YES	YES	YES	YES	YES	YES	YES	YES
Confounding factors identified	YES	NO	NO	YES	YES	YES	NO	YES	YES	YES	YES
Confounding factors accounted	YES	YES	NO	YES	YES	YES	YES	YES	YES	YES	YES
Follow-up complete enough	YES	YES	can't tell	YES	YES	YES	can't tell	YES	YES	YES	YES
Follow-up long enough	YES	YES	NO	can't tell	YES	YES	can't tell	YES	can't tell	YES	YES
What are the results of this study	OS,PFS,AEs	OS,PFS,AEs	OS,PFS,AEs	OS,PFS,AEs	OS,PFS,AEs	OS,PFS,AEs	OS,PFS,AEs	OS,PFS,AEs	OS,PFS,AEs	OS,PFS,AEs,OR	OS,PFS,AEs
Precise statistical results presented	YES	YES	YES	YES	YES	YES	YES	YES	YES	YES	YES
Believable results	YES	YES	YES	YES	YES	YES	YES	YES	YES	YES	YES
Results can be applied to the local population	YES	YES	YES	YES	YES	YES	YES	YES	YES	YES	YES
Results fit with other available evidence	YES	YES	YES	YES	YES	YES	YES	YES	YES	YES	YES
Recommendations for practice	YES	YES	YES	YES	YES	YES	YES	YES	YES	YES	YES
